# Multiple Micronutrients and Docosahexaenoic Acid Supplementation during Pregnancy: A Randomized Controlled Study

**DOI:** 10.3390/nu12082432

**Published:** 2020-08-13

**Authors:** Maddalena Massari, Chiara Novielli, Chiara Mandò, Stefania Di Francesco, Matteo Della Porta, Roberta Cazzola, Mauro Panteghini, Valeria Savasi, Silvia Maggini, Ella Schaefer, Irene Cetin

**Affiliations:** 1 Department of Woman, Mother and Neonate, Buzzi Children Hospital, ASST Fatebenefratelli Sacco, 20154 Milan, Italy; massari.maddalena@asst-fbf-sacco.it (M.M.); stefidfr@gmail.com (S.D.F.); irene.cetin@unimi.it (I.C.); 2 Department of Woman, Mother and Neonate, Luigi Sacco Hospital, ASST Fatebenefratelli Sacco, 20157 Milan, Italy; valeria.savasi@unimi.it; 3 “Luigi Sacco” Department of Biomedical and Clinical Sciences, Università Degli Studi di Milano, 20157 Milan, Italy; chiara.novielli@unimi.it (C.N.); chiara.mando@unimi.it (C.M.); matteo.dellaporta@unimi.it (M.D.P.); roberta.cazzola@unimi.it (R.C.); mauro.panteghini@unimi.it (M.P.); 4 Bayer Consumer Care AG, 4002 Basel, Switzerland; silvia.maggini@bayer.com

**Keywords:** docosahexaenoic acid, long-chain polyunsaturated fatty acids, maternal biomarkers, micronutrients, neurodevelopment, pregnant women, supplementation, vitamin D

## Abstract

Maternal dietary intake during pregnancy needs to meet increased nutritional demands to maintain metabolism and to support fetal development. Docosahexaenoic acid (DHA) is essential for fetal neuro-/visual development and in immunomodulation, accumulating rapidly within the developing brain and central nervous system. Levels available to the fetus are governed by the maternal diet. In this multicenter, parallel, randomized controlled trial, we evaluated once-daily supplementation with multiple micronutrients and DHA (i.e., multiple micronutrient supplementation, MMS) on maternal biomarkers and infant anthropometric parameters during the second and third trimesters of pregnancy compared with no supplementation. Primary efficacy endpoint: change in maternal red blood cell (RBC) DHA (*wt*% total fatty acids) during the study. Secondary variables: other biomarkers of fatty acid and oxidative status, vitamin D, and infant anthropometric parameters at delivery. Supplementation significantly increased RBC DHA levels, the omega-3 index, and vitamin D levels. Subscapular skinfold thickness was significantly greater with MMS in infants. Safety outcomes were comparable between groups. This first randomized controlled trial of supplementation with multiple micronutrients and DHA in pregnant women indicated that MMS significantly improved maternal DHA and vitamin D status in an industrialized setting—an important finding considering the essential roles of DHA and vitamin D.

## 1. Introduction

During pregnancy, an adequate maternal dietary intake is essential to meet the increased nutritional demands required to maintain metabolism and support fetal development [1]. Micronutrients such as folic acid and other B vitamins, vitamin D, vitamin C, calcium, copper, magnesium, iodine, selenium, zinc, and iron all have vital roles throughout all stages of pregnancy [2,3,4]. Poor dietary intake or deficiencies in both micro- and macronutrients can have adverse effects on pregnancy outcomes and neonatal health [5], including an increased risk of neural tube defects, preeclampsia, miscarriage, and low birth weight [6,7]. Many women are at risk of insufficient nutrient intake in industrialized as well as developing countries [8,9,10]. Therefore, micronutrient supplementation is frequently recommended during pregnancy to help improve pregnancy outcomes in the mother and child [11,12]. International guidelines (i.e., from the World Health Organization) currently recommend supplementation of iron and folic acid (0.4 mg/day) during the whole pregnancy for the purpose of improving pregnancy outcomes and for reducing maternal anemia in pregnancy [13]. Recently, there have been extensive scientific and medical discussions around the need to include vitamin D as a standard nutrient to be supplemented during pregnancy, due to low intake. Vitamin D regulates calcium and phosphate body stores and is therefore critical for bone health [14]. Furthermore, low concentrations of blood vitamin D in pregnant women have been associated with pregnancy complications [15,16].

In addition to micronutrients, a balanced macronutrient intake is recommended. In particular, the long-chain polyunsaturated fatty acids (LCPUFAs) found at high concentrations within the brain and central nervous system are essential for the development of the fetal brain [17]. Docosahexaenoic acid (DHA)—representing the largest proportion of LCPUFAs in the brain and retina—plays a key role during the pre- and early postnatal period [17,18,19,20]. After the first trimester, when the neural tube has closed and grey matter begins to form [21], DHA begins to rapidly accumulate in the brain [18,22]; accumulation continues for up to two years [23,24].

However, the human body is not efficient at producing essential LCPUFAs [22], and maternal concentrations decrease over the course of gestation [25]. Of note, the levels of DHA available to the fetus during pregnancy are governed by the diet of the mother [17,26,27,28]. Studies suggest that consumption of a diet rich in omega-3 LCPUFAs including DHA may have a reduced risk of common pregnancy complications such as intrauterine growth restriction, preeclampsia, and preterm deliveries [29,30,31]. Supplementation with DHA can also increase the expression of fatty acid transport proteins, thus increasing transport through the placenta and improving the fatty acid status of both the mother and child [32,33].

Meta-analyses have demonstrated that there are clinical benefits associated with prenatal multiple micronutrient [34] and LCPUFA supplementation [35] during pregnancy. However, there is limited data on the effects of prenatal supplementation in industrialized countries, particularly when used in combination. Clinical guidelines for pregnant women tend to focus on single nutrients for supplementation [36,37]. Given the interest in the potential beneficial effects of supplementation with micronutrients and DHA during pregnancy, we carried out a randomized trial to evaluate the effects of multiple micronutrients plus DHA supplementation during the second and third trimesters of pregnancy on maternal biomarkers compared with no supplementation in the control group in an industrialized country. The primary variable, i.e., the concentration of DHA (weight percent of total fatty acids (*wt*% TFA)) in maternal red blood cells (RBC), was considered indicative of LCPUFA status. Secondary explorative variables were other biomarkers of fatty acid and oxidative status, vitamin D, and anthropometric parameters of infants at delivery. We included vitamin D status as a secondary endpoint to investigate whether vitamin D supplementation is needed to maintain adequate status, and whether the levels of vitamin D in the supplement would be sufficient to maintain an adequate status. We hypothesized that supplementation might help to improve maternal DHA and vitamin D status in a healthy population of pregnant women, whereas dietary intake would be insufficient to meet the increased needs during pregnancy.

## 2. Materials and Methods

### 2.1. Trial Design

**Figure 1:**
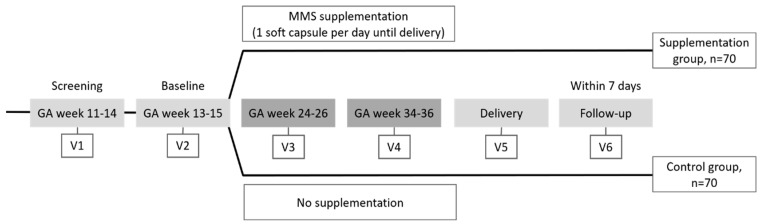
Study design. Visit 1 (V1, screening): pregnant women were screened for study eligibility and blood collection was performed. Visit 2 (V2, baseline): eligible women meeting the inclusion and exclusion criteria were randomized equally to one of the two study groups; nutritional status was assessed using a semi-quantitative FFQ. Visits 3 and 4 (V3 &V4, MMS supplementation or no supplementation): FFQ was administered and blood sampling took place—the red blood cell DHA level measured at Visit 4 was compared with the value measured at Visit 1 to assess the primary endpoint. Visit 5 (V5, delivery): obstetric evaluations were performed in all women and infant anthropometric parameters were measured. Concomitant medications and adverse events were assessed at all Visits. GA, gestational age; DHA, docosahexaenoic acid; FFQ, food frequency questionnaire; MMS, multiple micronutrients and DHA supplementation.

This was a multicenter, parallel, randomized controlled trial conducted at two centers in Italy to compare the effects of once daily supplementation with multiple micronutrients plus DHA (hereafter referred to as multiple micronutrient supplementation, or MMS) versus no supplementation during pregnancy on maternal biomarkers and infant anthropometric parameters. Supplementation began at gestational week 13–15 until delivery. Six visits were conducted during the trial, from screening to final follow-up, as outlined in Figure 1 and Supplementary Table S1. At baseline (Visit 2; gestational week 13–15), women who fulfilled the eligibility criteria were randomized to the supplementation or control group in a 1:1 ratio. The sequential randomization list (generated through a validated SAS program by an independent statistician) was generated according to permutated block codes. A randomization number was assigned to each woman at each site by means of randomization cards. The study was not blinded. All blood parameters were measured at Visits 1, 3, and 4 in all women, while dietary intake was recorded at Visits 2, 3, and 4.

The study was approved by an independent ethics committee (Comitato Etico Milano, Milan, Italy). The Institutional Review board Project no. of the study: 2016/ST/024. The study was approved on 30 March 2016. The study was conducted in accordance with the Declaration of Helsinki and in compliance with all current Good Clinical Practice guidelines, local laws, regulations, and organizations. The trial was registered at ClinicalTrials.gov (ClinicalTrials.gov Identifier: NCT04438928). The trial protocol can be obtained from the corresponding author, upon reasonable request.

### 2.2. Study Population

Healthy, pregnant Caucasian women aged 18–42 years were screened during their first trimester prenatal visit (gestational age (GA), week 11–14) at Hospital Sacco and Hospital Buzzi in Milan, Italy. The study was proposed to all pregnant women with a singleton pregnancy within the gestational age indicated. Women were included in the study if they were having a singleton pregnancy, hemoglobin level >105 g/L, normal ultrasound examination, and inconspicuous fetal anomaly screening, taking at least 400 μg folate per day, and provided written, signed informed consent for participation in the study. Women were excluded if they had experienced previous adverse pregnancy outcomes, followed a specific diet, or were already taking DHA/multivitamin supplements (except folate or iron). Full inclusion and exclusion criteria are listed in Supplementary Table S2.

### 2.3. Study Product

The study product was an oral MMS soft gel capsule (Elevit, Bayer) that contained 12 vitamins, six minerals, and DHA (200 mg) to meet the requirements of women during pregnancy, especially during the second and third trimester [38,39] (Supplementary Table S3). One capsule was taken per day with a sufficient amount of liquid, from GA week 13–15 (Visit 2, baseline) until delivery (Visit 5; approximately 27 weeks of supplementation). The control group did not receive a placebo during this time.

### 2.4. Parameters Assessed

Analyses were performed at the “Luigi Sacco” Department of Biomedical and Clinical Sciences (Università degli Studi di Milano) and ASST Fatebenefratelli Sacco, Milan, Italy. In total, approximately 56 mL of blood was taken in the fasted state from each subject for the efficacy and safety assessments during the whole study. Blood samples were centrifuged for 10 min at 1000 g at 4 °C; plasma for 8-isoprostane and dROMs analysis was separated from the erythrocyte pellet, and the buffy coat was discarded. Erythrocytes for fatty acid and glutathione analyses were washed once with a 0.2 M EDTA + 150 nM NaCl solution through gentle inversion, and then 15 min centrifugation at 2000 g at 4 °C.

The efficacy parameters assessed are outlined in Supplementary Table S4. The change in RBC DHA (*wt*% TFA) from Visit 1 to Visit 4 was the primary maternal variable to assess the beneficial effects of supplementation with micronutrients and DHA during the second and third trimesters of pregnancy. Secondary maternal variables included other RBC fatty acid parameters (TFA, eicosapentaenoic acid (EPA), *wt*% TFA, DHA/TFA ratio, and omega-3 index), calcidiol (25-hydroxyvitamin D), and oxidative stress markers in blood including reduced glutathione (GSH)/oxidized glutathione (GSSG) ratio, plasma reactive oxygen metabolites (ROMs, which are hydroperoxides), and plasma 8-isoprostane. The erythrocyte membrane fatty acid composition was determined by gas chromatography of fatty acid methyl esters [40,41,42]. The amount of each considered fatty acid was calculated as µg/mL of RBCs and expressed as a percentage of the total fatty acid concentration. The omega-3 index was calculated by summing the percentage of EPA and DHA [43]. Calcidiol levels were measured using radioimmunoassay [44], the GSH/GSSG ratio using fluorimetric assay [1], ROMs using photometric assay [45,46], and 8-isoprostane using competitive enzyme immunoassay with an ELISA kit (Cayman Chemical, Ann Arbor, MI, USA) following the manufacturer’s instructions [47]. Briefly, 500 uL of heparinated plasma were stored at –80 °C with a preservative ethanol solution containing butylated hydroxytoluene (BHT) until analysis. Alkaline hydrolysis was performed to allow total 8-isoprostane (both free and esterified fractions) quantification; after neutralization, proteins were removed by ethanol precipitation and samples were purified by solid phase extraction (SPE) using octadecyl (C-18) silica affinity cartridges. Total 8-isoprostane levels in purified plasma samples were then analyzed by ELISA. Dietary intake was evaluated using a semi-quantitative Food Frequency Questionnaire of five food categories to assess the usual daily intake of foods and nutrients (adapted from Vioque et al. [48], which was validated in pregnant women) at Visits 2, 3, and 4. Dietary intake data and results of a small subgroup analysis in women who underwent a cesarean section (cord blood and placenta samples) will be presented elsewhere.

Safety and tolerability were assessed by evaluating the incidence and severity of adverse events (AEs) and their relationship to trial treatment. Laboratory parameters, physical examination, and vital signs were also recorded.

### 2.5. Statistical Analysis

Assuming a treatment difference of 1.6 (standard deviation (SD) 3.4), as observed by Bergmann et al. 2008 [49], 70 subjects per arm were required to achieve 80% power with 0.05 of alpha to detect the treatment difference between the supplementation and control groups. To account for a drop-out rate of 15%, approximately 164 subjects (82 per treatment group) were to be randomized to get 140 evaluable subjects.

The primary efficacy analysis was performed on the per protocol (PP) population (all subjects with efficacy data for the primary efficacy endpoint at Visit 4 who did not have protocol violations). Results were corroborated using data from the intent-to-treat (ITT) population (i.e., all subjects in the safety population who had at least one post-baseline measurement of efficacy data). The safety population comprised all subjects who were randomized into the study, and took at least one dose of the supplement for those randomized to the treatment group. 

The primary efficacy endpoint was defined as the change in maternal RBC DHA (*wt*% total fatty acids) from Visit 1 to Visit 4, analyzed using the analysis of covariance (ANCOVA) with treatment as a fixed effect and the Visit 1 value as covariate. Secondary maternal efficacy endpoints were changes from Visit 1 to Visit 4 in blood fatty acid parameters (RBC EPA (*wt*% total fatty acids), DHA/EPA ratio, RBC omega-3 index), 25-hydroxyvitamin D, and antioxidant status (GSH/GSSG ratio, plasma ROMs, 8-isoprostane). All secondary endpoints were analyzed similarly to the primary endpoint. Secondary infant efficacy endpoints (gestational age, head circumference, weight and length measurements, ponderal index, infant skinfold thickness, Apgar score, bone density) were collected at delivery (Visit 5) or within 10 days after delivery for bone density and analyzed using ANCOVA with treatment as fixed effect.

Safety and tolerability variables were assessed by evaluating incidence and severity of AEs, their relationship to trial treatment, and the incidence of abnormal findings in measurement of objective tolerability through vital signs, physical examination, and clinical laboratory findings. Only treatment-emergent AEs (TEAEs) were analyzed, i.e., AEs that began or worsened after randomization.

Two-sided *p*-values < 0.05 were considered statistically significant. Results are presented as mean ± standard deviation (range), *n* (%), or LSMEANS (least squares means) of change from Visit 1 (95% confidence interval, CI), as appropriate. All statistical tables, listings, and analyses were produced using SAS^®^ release 9.4 or later (SAS Institute, Inc., Cary, NC, USA). 

## 3. Results

### 3.1. Subject Characteristics

**Figure 2:**
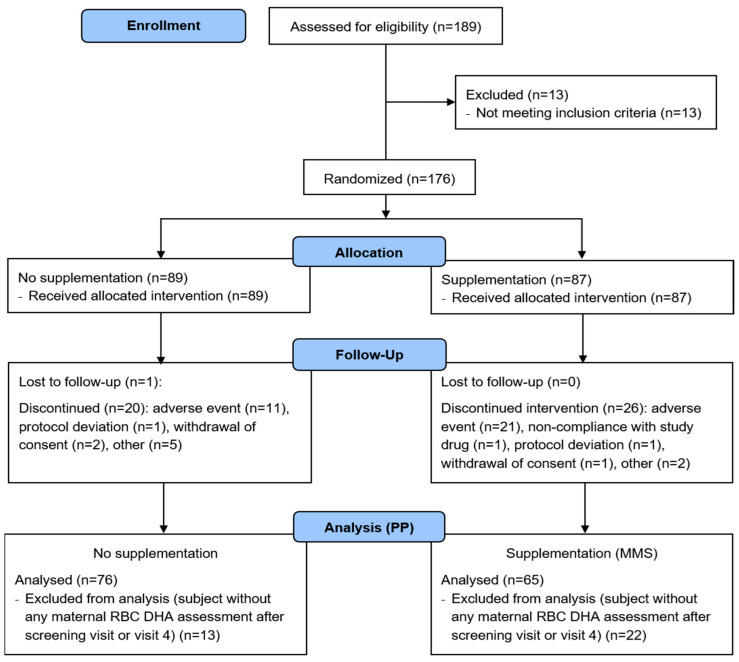
Flow diagram for study participants. DHA, docosahexaenoic acid; MMS, multiple micronutrients and DHA supplementation; RBC, red blood cells; PP, per protocol.

The study took place between September 2016 to December 2019. After screening, 176 subjects were randomized to the MMS (*n* = 87) or control (*n* = 89) groups (Figure 2). All subjects were included in the safety population. Forty-six subjects discontinued the study, mainly because of adverse events (32 (69.6%) subjects). The PP population comprised 141 subjects (MMS, *n* = 65; control, *n* = 76). The mean study duration was 24.5 ± 6.49 (1.0–30.9) weeks, and was comparable in both groups. Overall compliance was ≥80% in 63 (72.4%) of MMS subjects, ≤80% in four (4.6%), and unknown in 20 (23%).

Subject baseline demographics, clinical characteristics, and delivery information are shown in Table 1. The mean age was 31.9 ± 4.64 (18–41) years and all subjects were Caucasian. All demographics were similar between groups, with no significant differences. No abnormalities in physical or gynecological examinations were reported at Visit 1 or Visit 2. Although not statistically significant, a higher proportion of subjects in the control group compared with the MMS group experienced delivery complications (16 (23.2%) vs. eight (12.9%) subjects, respectively) or had an induced labor (13 (18.8%) vs. nine (14.5%) subjects). The groups were well balanced regarding infant sex (male 58.1% in the MMS group, 56.5% in the control group).

### 3.2. Efficacy Endpoints

**Figure 3:**
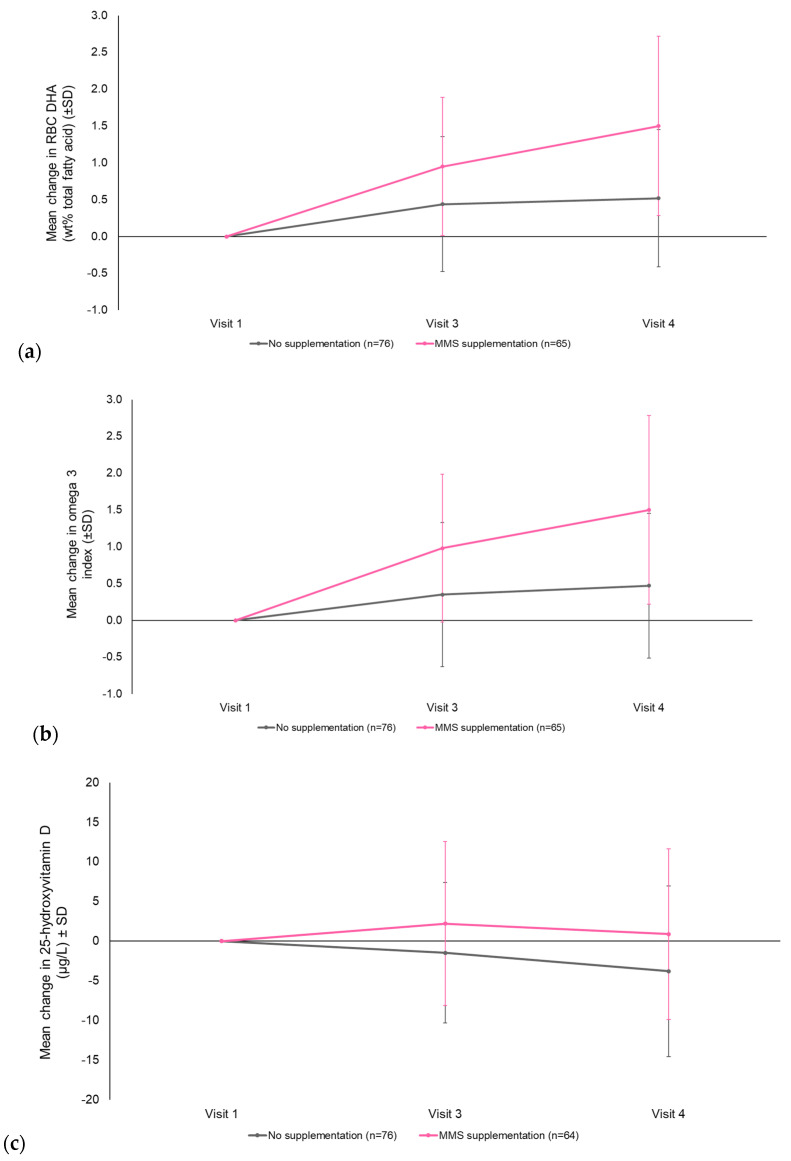
Mean change (± standard deviation) from Visit 1 to Visit 4 in maternal (**a**) RBC DHA (*wt*% TFA) (*p* < 0.0001 in favor of MMS), (**b**) omega 3 index (*p* < 0.0001 in favor of MMS), and (**c**) calcidiol (25-hydroxyvitamin D) (*p* = 0.0122 in favor of MMS) (per protocol population; LOCF approach). Visit 1: Screening (GA Week 11/14); Visit 3: GA Week 24/26; Visit 4: GA Week 34/36. DHA, docosahexaenoic acid; GA, gestational age; LOCF, last observation carried forward; MMS, multiple micronutrients and DHA supplementation; RBC, red blood cells; SD, standard deviation; TFA, total fatty acids; *wt*, weight.

**Primary.** Maternal RBC DHA (*wt*% TFA) increased every visit in both groups (Figure 3 and Table 2), but the mean change from Visit 1 to Visit 4 was significantly greater in the MMS group compared with the control group, with an estimated treatment difference of 0.96 (95% CI 0.61, 1.31) (*p* < 0.0001) (Table 2). Furthermore, RBC DHA levels in women at the lower ranges increased by a greater extent in the MMS group (1.1% at Visit 3 and 1.6% at Visit 4 vs. Visit 1) compared to those in the control group (increase of 0.2% at Visit 3 and 0.5% at Visit 4 vs. Visit 1), and reached threshold levels (5% [50]) by Visit 4 (Table 2).

**Secondary maternal endpoints.** Significant differences were observed in favor of MMS for maternal RBC DHA/TFA ratio (estimated difference 0.01 (95% CI 0.006, 0.013); *p* < 0.0001), omega-3 index (estimated difference 1.00 (95% CI 0.64, 1.37); *p* < 0.0001), and calcidiol (estimated difference 3.96 (95% CI 0.88, 7.04) μg/L; *p* = 0.0122) (Figure 3 and Table 2). 

The remaining secondary efficacy endpoints (maternal RBC TFA, RBC EPA (*wt*% TFA, GSH/GSSG ratio, ROMs, 8-isoprostane)) were comparable between groups, albeit slightly higher in the MMS group, with no significant differences (Supplementary Table S5).

**Secondary infant endpoints.** As outlined in Supplementary Table S6, infant variables were comparable between groups, with no statistically significant differences apart from subscapular skinfold thickness (thicker in the MMS group, *p* = 0.0292) and bone density in m^2^ (borderline significantly greater in the control group, *p* = 0.0486).

**Dietary intake.** Assessment of dietary intake showed that consumption of the macro- and micronutrients measured was comparable between groups at each visit (Supplementary Table S7). 

### 3.3. Safety Analysis

As outlined in Table 3, 125 (71.0%) subjects reported at least one TEAE pertinent to the mother (232 TEAEs overall) and 23 (13.1%) subjects reported them as serious, with a comparable number in each group. In the MMS group, 19 (21.8%) had one TEAE that led to permanent treatment discontinuation. Only three (3.5%) subjects in the MMS group had at least one suspected related TEAE (vomiting, with mild severity). At least one TEAE pertinent to the fetus/child were reported in ten (5.7%) subjects (13 TEAEs overall), and five (2.8%) reported them as serious. A higher proportion of subjects reported a TEAE in the MMS group, but none were considered to be treatment related. One (1.6%) subject had one TEAE pertinent to the fetus/child that led to permanent discontinuation. There was one fatality in the MMS group unrelated to study treatment. No relevant changes in clinical laboratory parameters (i.e., hematology, kidney function, liver function, blood coagulation, CRP) were observed, although there was a decrease in mean ferritin levels in both groups over the course of the study. Physical and gynecological examinations were normal throughout. 

## 4. Discussion

Supplementation with MMS plus DHA throughout the second and third trimester of pregnancy led to a significant increase in RBC levels of DHA, as well as the proportion of DHA compared with EPA and TFA. There was also a significant increase in the omega-3 index, while vitamin D levels increased during the course of the study compared to a decrease in women who did not receive supplementation. In the infant, a significantly greater subscapular skinfold thickness was observed in the MMS group. Safety outcomes were comparable between groups and MMS was well tolerated.

Our findings demonstrate that RBC DHA levels were significantly higher in the MMS group than in the control group. In pregnant women, the target RBC DHA level is 5% [50] (with <4.3% considered very low [51]). In our study, although average RBC DHA levels were above 6% at each visit (with higher levels in the MMS group), the lower ranges indicated that some women in both groups fell below this value. Nevertheless, RBC DHA levels in women at the lower ranges increased by a greater extent in the MMS group compared to those in the control group over the course of the study, and reached the threshold by the third trimester (Table 2).

The omega-3 index was also significantly higher after supplementation. As RBC EPA values were comparable between groups, the increase in omega-3 index must be the result of an increase in DHA. In cardiovascular disease, the target range for the omega-3 index is 8–11%; it has been suggested that this range might also be suitable during pregnancy and lactation [52]. Reference values of 7.5–10.0% have also been recommended in pregnant women [53]. In our study, while the omega-3 index increased from 6.7% to 7.1% in the control group, the increase was greater (6.5% to 8.0%) in the MMS group. Therefore, supplementation with DHA helped women to reach target levels during pregnancy. 

Current nutritional recommendations indicate that pregnant and lactating women should aim to achieve an average dietary intake of at least 200 mg DHA/day [54]. However, consumption of omega-3 fatty acids remains low particularly in pregnant and lactating women [55]. This is of relevance considering the vital roles of DHA in neurodevelopment, visual development, and neuroinflammation [56]. Moreover, pregnancy syndromes such as gestational diabetes and preeclampsia have also been associated with altered maternal omega-3 status and placental omega-3 metabolism [57,58,59].

The finding that there was a significant increase in calcidiol levels in supplemented women, but not in the non-supplemented control group, is also of interest. Vitamin D is essential for the health of both the developing fetus and the mother [60], and insufficient levels may have an adverse effect on skeletal homeostasis in the infant [61] and increase the maternal risk of preeclampsia [5].

In our study, no significant differences were observed between supplemented and control women regarding the markers of oxidative status. Oxidative stress has been implicated in many pathological processes during pregnancy [5]. However, this particular population of pregnant women was selectively chosen as a low-risk population, likely not at risk for decreased antioxidant status. Moreover, the sample size of the study was calculated based on the primary outcome; therefore, these results must be considered exploratory.

To our knowledge, this is the first randomized controlled trial evaluating the combination of MMS plus DHA in pregnant women. Our results indicate that in a high-income country setting, supplementation with micronutrients in combination with DHA can optimize maternal DHA status [49,62,63], despite the women in our supplemented group having a slightly lower intake of DHA from food. The timing of supplementation is important, and should occur in line with the development and growth of the embryonic brain, particularly during the later stages of pregnancy [17,21] when DHA rapidly begins to accumulate [18,22]. Furthermore, supplementation with MMS during pregnancy, as in our study, can improve maternal and infant outcomes, leading to reductions in the incidence of pre-eclampsia [64], neural-tube defects [64,65], low birthweight and small-for-gestational age babies [3], limb reduction defects, and congenital urinary tract abnormalities [64]. There may also be long-term benefits in children [4] (e.g., cognitive development [66,67]). Although many of these results have been reported from low- to middle-income countries, micronutrient levels in pregnant women are often insufficient even in industrialized countries, where dietary resources are more readily available [12]. However, the routine use of multivitamins during pregnancy has not yet been recommended in high-income countries, despite the benefits on clinical outcomes [68]. Currently, only folic acid and iron are recommended as standard interventions in pregnancy in industrialized countries [37].

Further research is necessary to better understand whether the improvements in maternal DHA status, as well as other improvements in omega-3 index and calcidiol levels, have a positive impact on maternal and infant clinical outcomes. Large, long-term randomized controlled trials on MMS supplementation including DHA are essential.

Our study has some limitations, including the lack of a placebo control group and the consequent unblinded nature of the study (which could have led to expectation bias [69]), the small sample size, and the fact that only Caucasian women were included (which limits the generalizability of the results). Adequately powered studies with a varied study population are necessary to better establish the impact of different baseline characteristics in pregnant women and to evaluate clinical outcomes.

## 5. Conclusions

Supplementation with MMS plus DHA in pregnant women can complement dietary intake and significantly improve maternal DHA and vitamin D status. This finding is important in light of the essential roles of DHA in the developing brain of the fetus, in visual development, and in immunomodulation.

## Figures and Tables

**Table 1:** Subject characteristics at baseline (values expressed as *n*, mean ± standard deviation, and median (range), unless otherwise stated) and delivery information (values expressed as *n* (%), unless otherwise stated) (per protocol population).

Characteristics	No Supplementation (*n* = 76)	MMS (*n* = 65)
Age (years)	76	65
	32.3 ± 4.72	31.4 ± 4.52
	33.0 (18–41)	32.0 (20–40)
Weight (kg)	76	65
	61.5 ± 9.96	63.2 ± 9.48
	59.0 (45–87)	47.0 (47–95)
Height (cm)	76	65
	164.1 ± 7.08	165.9 ± 5.60
	165.0 (147–184)	165.0 (150–178)
Body mass index (kg/m^2^)	76	65
	22.8 ± 3.24	22.9 ± 3.10
	21.7 (18.0–29.7)	22.0 (18.1–29.9)
Previous pregnancy, *n* (%)		
No	30 (39.5)	30 (46.2)
Yes	46 (60.5)	35 (53.9)
Smoking status, *n* (%)		
Never	49 (64.5)	49 (75.4)
Former *^a^*	27 (35.5)	16 (24.6)
Delivery information		
Subjects performing delivery visit	69	62
Type of delivery, *n* (%)		
Vaginal	55 (79.7)	49 (79.0)
Caesarean	14 (20.3)	13 (21.0)
Delivery complications, *n* (%)		
No	53 (76.8)	54 (87.1)
Yes	16 (23.2)	8 (12.9)
Induced labor, *n* (%)		
No	56 (81.2)	53 (85.5)
Yes	13 (18.8)	9 (14.5)
Infant sex, *n* (%)		
Male	39 (56.5)	36 (58.1)
Female	30 (43.5)	26 (41.9)

*^a^* Stopped smoking prior to pregnancy/when becoming aware of pregnancy consent signature plus one day. MMS, multiple micronutrients and docosahexaenoic acid supplementation.

**Table 2:** Primary and secondary outcomes at each visit (LOCF approach; values presented as mean ± standard deviation (range)) and differences between groups from Visit 1 to Visit 4 (presented as LSMEANS (95% confidence interval), ANCOVA model) (per protocol population).

	No Supplementation (*n* = 76)			MMS (*n* = 65)		
	Visit 1	Visit 3	Visit 4	Visit 1	Visit 3	Visit 4
RBC DHA (*wt*% TFA)	6.1 ± 1.23 (3.8–9.3)	6.6 ± 1.30 (4.0–10.4)	6.7 ± 1.34 (4.3–9.6)	6.1 ± 1.26 (3.4–10.2)	7.0 ± 1.30 (4.5–10.5)	7.5 ± 1.48 (5.0–13.0)
LSMEANS difference/*p* value	—	—	—	—	—	0.96 (0.61, 1.31)/ < 0.0001 *
RBC DHA/TFA ratio	0.06 ± 0.01 (0.04–0.09)	0.07 ± 0.01 (0.04–0.10)	0.07 ± 0.01 (0.04–0.10)	0.06 ± 0.01 (0.03–0.10)	0.07 ± 0.01 (0.04–0.11)	0.08 ± 0.01 (0.05–0.13)
LSMEANS difference/*p* value	—	—	—	—	—	0.010 (0.006, 0.013)/ <0.0001 *
Omega 3 index (%)	6.7 ± 1.38 (4.2–10.1)	7.0 ± 1.43 (4.2–10.7)	7.1 ± 1.45 (4.5–10.0)	6.5 ± 1.40 (3.7–10.9)	7.5 ± 1.43 (4.7–11.1)	8.0 ± 1.59 (5.3–13.6)
LSMEANS difference/*p* value	—	—	—	—	—	1.00 (0.64, 1.37)/ <0.0001 *
Calcidiol (ug/L)	21.6 ± 8.94 (5.5–48.8)	19.9 ± 9.87 (4.6–64.1)	17.8 ± 9.72 (4.0–45.0)	20.5 ± 7.54 (4.4–36.5)	22.8 ± 8.94 (4.0–48.6)	21.4 ± 9.07 (5.5–42.7)
LSMEANS difference/*p* value	—	—	—	—	—	3.96 (0.88, 7.04)/ 0.0122 *

* Two-sided *p* value <0.05 considered statistically significant. Visit 1: Screening (GA Week 11/14); Visit 3: GA Week 24/26; Visit 4: GA Week 34/36. DHA, docosahexaenoic acid; GA, gestational age; LOCF, last observation carried forward; LSMEANS, least squares means (difference = supplementation—no supplementation); MMS, multiple micronutrients and DHA supplementation; RBC, red blood cells; TFA, total fatty acids; wt, weight.

**Table 3:** Summary of participants with treatment-emergent adverse event (safety population; values expressed as *n* (%) subjects).

Parameters	No Supplementation (*n* = 89)	MMS (*n* = 87)	Total (*n* = 176)
Number of TEAEs pertinent to the mother	114	118	232
Any TEAEs pertinent to the mother	64 (71.9)	61 (70.1)	125 (71.0)
At least one suspected related *^a^*	NA	3 (3.5)	3 (1.7)
At least one serious TEAE	11 (12.4)	12 (13.8)	23 (13.1)
At least one leading to temporary treatment interruption *^b^*	NA	1 (1.2)	1 (0.6)
At least one leading to permanent treatment discontinuation *^c^*	NA	19 (21.8)	19 (10.8)
Fatal outcome	0	0	0
Number of TEAEs pertinent to fetus/child	4	9	13
Any TEAEs pertinent to fetus/child	3 (3.4)	7 (8.1)	10 (5.7)
At least one suspected related *^a^*	NA	0	0
At least one serious TEAE *^b^*	2 (2.3)	3 (3.5)	5 (2.8)
At least one leading to temporary treatment interruption *^c^*	NA	1 (1.2)	1 (0.6)
At least one leading to permanent treatment discontinuation *^d^*	NA	1 (1.2)	1 (0.6)
Fatal outcome	0	1 (1.2)	1 (0.6)

*^a^* Suspected related adverse events were those events with causal relationship equal to related; *^b^* No Supplementation group, the TEAEs pertinent to the fetus/child classified as severe were: fetal distress syndrome 1 (1.12%), fetal growth restriction 1 (1.12%); MMS group, the TEAEs pertinent to the fetus/child classified as severe were “Duodenal atresia” (1, 1.15%), “Fetal compartment fluid collection “(1, 1.15%), “Fetal growth restriction” (1, 1.15%) and “Polyhydramnios” (1, 1.15%). No TEAE pertinent to the fetus/child was suspected of being related to the study product; *^c^* adverse events leading to temporary treatment interruption were those events with action taken equal to drug interrupted; *^d^* adverse events leading to permanent treatment discontinuation were those events with action taken equal to drugs withdrawn. MMS, multiple micronutrients and docosahexaenoic acid supplementation; NA, not applicable; TEAEs, treatment-emergent adverse events.

## Supplementary Materials

The following are available online at https://www.mdpi.com/2072-6643/12/8/2432/s1, Table S1: Assessment schedule, Table S2: Full list of inclusion and exclusion criteria, Table S3: Composition of the multimicronutrient supplement (MMS) compared to the recommended dietary allowance (RDA) and upper tolerable limits (UL) for pregnant women, Table S4: Blood and plasma sampling for efficacy parameters in all pregnant women, Table S5: Change in primary and secondary maternal efficacy endpoints from Visit 1 to Visit 4 (gestational age week 34/36), Table S6: Infant assessments, Table S7: Daily macronutrient intakes during the study (per protocol population) compared with recommended allowances for pregnant women.
