# Supplementary material for: Multiple Micronutrients and Docosahexaenoic Acid Supplementation during Pregnancy: A Randomized Controlled Study
Source: Nutrients. 2020 Aug 13;12(8):2432. doi: 10.3390/nu12082432 (PMC7468952; doi:10.3390/nu12082432)
Supplement: Supplementary file 1 [file nutrients-12-02432-s001.pdf]

Supplementary tables

Table S1. Assessment schedule.

Visit (timing)	V1 (screening: GA wk 11-14)	V2 (baseline: GA wk 13-15)	V3 (GA wk 24-26)	V4 (GA wk 34-36 or early discontinuation)	V5 (delivery)	V6 (follow-up: within 7 days after delivery)
Subject informed consent	X					
Inclusion/exclusion criteria	X	X				
Medical/surgical history	X					
Demographics	X					
History of previous birth outcomes	X					
History of folate and iron/multimicronutrient intake	X					
History and review of drug, alcohol and nicotine use	X	X	X	X	X	
Assessment of pregnancy	X					
Assessment if elective Caesarean section was planned or recommended	X					
Medication history over past 30 days	X					
Physical examination including gynecological examination	X	X	X	X	X	X
Vital signs (sitting blood pressure, pulse rate)	X	X	X	X	X	X
Height, weight and body mass index	X			X		
Type of delivery					X	
Gestational length					X	
Induced labor (need for urgent delivery)					X	
Delivery complications					X	
Blood sampling for RBC fatty acid parameters (TFA, DHA wt% TFA, EPA wt% TFA, DHA/TFA ratio, omega 3 index)	X		X	X	X [a]	

Visit (timing)	V1 (screening: GA wk 11-14)	V2 (baseline: GA wk 13-15)	V3 (GA wk 24-26)	V4 (GA wk 34-36 or early discontinuation)	V5 (delivery)	V6 (follow-up: within 7 days after delivery)
Calcidiol (25-hydroxyvitamin D)	X		X	X	X [a]	
Oxidative status: GSH/GSSG ratio, plasma lipid hydroperoxides (ROMs), 8-isoprostane	X		X	X	X [a]	
Randomization of supplementation kit to subject		X				
Check for supplementation compliance			X	X	X	
Supplementation		X [b]	X [b]	X [b]		
Dispensing of study treatment		X	X	X		
Return of study treatment			X	X	X	
Review of concomitant medications		X	X	X	X	
Food consumption assessment (Food Frequency Questionnaire)		X	X	X		
Infant assessments [c]					X	
Cord blood evaluations [a]					X	
Placental evaluations [a]					X	
Assess adverse events		X	X	X	X	X
Safety laboratory	X [d]	X [e]	X [e]	X		
Subject identification assignment	X					
Subject discontinuation				(X)		

[a] Only in a subset of women (approximately 10 per study group) undergoing elective caesarean section. [b] Once daily administration of one soft gel capsule of the multimicronutrient supplement, from GA wk 13-15 to delivery, in the supplementation group only (the control group did not receive any treatment or placebo). [c] Infant assessments include infant sex, gestational age, head circumference, weight and length measurements, ponderal index, infant skinfold thickness, Apgar score; bone density was assessed within 10 days after Visit 5. [d] At screening, additional samples for Hepatitis B and C and human immunodeficiency virus (HIV I and II) serology screening. [e] At Visits 2 and 3, only complete blood count was performed as safety assessment. DHA, docosahexaenoic acid; EPA, eicosapentaenoic acid; GA, gestational age; GHS, reduced glutathione; GSSG, oxidized glutathione; RBC, red blood cell; ROMs, reactive oxygen metabolites; TFA, total fatty acid; V, visit; wk, week; wt, weight.

Table S2. Full list of inclusion and exclusion criteria.

Inclusion criteria	1. Healthy pregnant Caucasian women aged 18 to 42 years (inclusive) in trimester 1-2 (gestational age week 11-14 at screening)<br>2. Omnivore diet<br>3. Intention to deliver in the study center<br>4. Hemoglobin >105 g/L<br>5. Inconspicuous fetal anomaly screening<br>6. Normal ultrasound examination (ultrasonography)<br>7. Singleton pregnancy<br>8. Taking at least 400 μg folate per day<br>9. Seronegative for human immunodeficiency virus, hepatitis B, and hepatitis C at screening<br>10. Not participating in any other study<br>11. Willing and able to participate in all scheduled visits, adhere to the supplementation plan, and to laboratory tests and to all other study related procedures<br>12. All women provided a signed and dated informed consent to participate in the study
Exclusion criteria	1. Physical (including vital signs, e.g., blood pressure, pulse rate), hematological and clinical-chemical parameters deviating from normal and with clinical relevance<br>2. Any infection (acute or chronic) at screening and baseline<br>3. Any current metabolic diseases (e.g. diabetes, hypothyroidism)<br>4. Less than 12 months from previous delivery<br>5. Any history or current diseases, associated with malabsorption, or other severe diseases of the gastrointestinal tract (e.g. chronic inflammatory bowel disease, iron accumulation, iron utilization disorders)<br>6. Any history or current neurological, cardiac, endocrine or bleeding disorders<br>7. Specific diets (e.g. vegan, vegetarian, celiac, lactose-free)<br>8. Body mass index <18 or >30 kg/m^2<br>9. Pregnant women already taking DHA/multivitamin supplements (except folate or iron) or intending to supplement during the study period<br>10. Diagnosed or suspected malignant or premalignant disease<br>11. Current clinically significant depression<br>12. Current intake of pharmaceuticals or dietary supplements which may interact with any of the ingredients of the trial treatment (i.e. fluoroquinolones, bisphosphonates, levodopa, levothyroxine, penicillamine, antibiotics containing tetracycline or trietine)<br>13. History of or current diseases where vitamin, mineral, trace element or DHA supplementation might be not recommended/contraindicated (e.g., sickle cell anemia, copper metabolism disorders (Wilson's disease), renal disease,

nephrolithiasis, urolithiasis, hypercalcemia, hypercalciuria, hepatobiliary diseases, existing hypervitaminosis, iron metabolism disorders, hypermagnesemia)

14. Severe hyperemesis gravidarum
15. Previous adverse birth outcomes (e.g. small for gestational age, low birth weight, premature birth, still-birth, more than two consecutive spontaneous abortions)
16. Previous adverse pregnancy outcomes (e.g. gestational diabetes)
17. Diagnosed congenital abnormalities in current or previous pregnancy
18. Known carrier or affected with a genetic disease or condition (e.g. mutation carrier for autosomal recessive diseases)
19. History of or current abuse of drugs, alcohol or other substances
20. Participation in another clinical trial within 30 days prior to screening (including nutritional studies)
21. Current smokers or women who smoked during current pregnancy
22. Any history of hypersensitivity or known allergy to any of the ingredients of the study supplement
23. Incapability of understanding the language in which the study related information was given
24. Close affiliation with the investigational site, e.g., a close relative of the investigator, dependent person (e.g. employee or student of the investigational department/unit)
25. Unwilling or unable to comply with all requirements outlined in the protocol
26. Women previously enrolled into the study could not be re-included

DHA, docosahexaenoic acid.

Table S3. Composition of the multimicronutrient supplement (MMS) compared to the recommended dietary allowance (RDA) and upper tolerable limits (UL) for pregnant women.

Micronutrient, units	MMS (Elevit, Bayer)	Institute of Medicine [1] [a]	
		RDA	UL
Vitamin A (beta carotene), IU	2566	2567 (2500)	10000 (9333)
Vitamin C, mg	85	85 (80)	2000 (1800)
Vitamin D, IU	200	600	4000
Vitamin E, IU	15	22.35	1490 (1192)
Vitamin B1, mg	1.4	1.4	ND
Vitamin B2, mg	1.4	1.4	ND
Vitamin B3, mg	18	18	35 (30)
Vitamin B5, mg	6	6	ND
Vitamin B6, mg	1.9	1.9	100 (80)
Folic acid, µg	200	—	—
Folic acid (as L-5-MTHF, Ca), µg	226	600	1000 (800)
Vitamin B12, µg	2.6	2.6	ND
Biotin, µg	30	30	ND
Iodine, µg	150	220	1100 (900)
Magnesium, mg	57	350-360	350
Zinc, mg	10	11	40 (34)
Selenium, µg	60	60	400
Copper, mg	1.0	1.0	10 (8)
Iron, mg	14	27	45
DHA, mg	200	250 + 100-200 [b]	—

[a] Specific recommendation for pregnant women, value in parentheses indicates specific recommendation for pregnant women <19 years old; [b] European Food Safety Authority recommendation of 250 mg/day EPA + DHA plus 100-200 mg/day DHA for pregnant and lactating women [2]. DHA, docosahexaenoic acid; MTHF Ca, methyltetrahydrofolate, calcium; MMS, multiple micronutrient supplementation; ND, not determined (insufficient scientific data in pregnant women).

Table S4. Blood and plasma sampling for efficacy parameters in all pregnant women.

Parameter	Sampling time point [a]
RBC TFA	Visits 1, 3, 4
RBC DHA wt% TFA	
RBC EPA wt% TFA	
RBC DHA/ TFA ratio	
ROMs	
8-isoprostane	
RBC omega 3 index	
25-hydroxyvitamin D [b]	
GSH/GSSG ratio	

[a] Visit 1 (screening): gestational age (GA) week 11-14; Visit 3: GA week 24-26; Visit 4: GA week 34-36. [b] Calcidiol, the major circulating form of vitamin D. DHA, docosahexaenoic acid; EPA, eicosapentaenoic acid; GSH, reduced glutathione; GSSG, oxidized glutathione; RBC, red blood cells; ROMs, reactive oxygen metabolites; TFA, total fatty acids; wt, weight.

Table S5. Change in primary and secondary maternal efficacy endpoints from Visit 1 to Visit 4 (gestational age week 34/36) (per protocol population; ANCOVA model – LOCF approach, values expressed as LSMEANS (95% confidence interval)).

Outcomes	No supplementation (N=76)	MMS (N=65)	Difference [a]	P value
Primary outcome				
RBC DHA (wt% TFA)	0.52 (0.28, 0.76)	1.48 (1.22, 1.74)	0.96 (0.61, 1.31)	<0.0001 *
Secondary outcomes [b]				
RBC TFA	-190.0 (-433.2, 53.2)	35.7 (-221.5, 293.0)	225.7 (-122.0, 573.4)	0.2014
RBC EPA (wt% TFA)	-0.04 (-0.10, 0.01)	-0.01 (-0.07, 0.05)	0.03 (-0.05, 0.11)	0.4291
RBC DHA/TFA ratio	0.005 (0.003, 0.008)	0.015 (0.012, 0.017)	0.010 (0.006, 0.013)	<0.0001 *
Omega 3 index (%)	0.47 (0.21, 0.72)	1.47 (1.20, 1.74)	1.00 (0.64, 1.37)	<0.0001 *
Calcidiol (ug/L)	-3.48 (-5.62, -1.33)	0.48 (-1.81, 2.77) [c]	3.96 (0.88, 7.04)	0.0122 *
GSH/GSSG ratio	0.46 (0.00, 0.92)	0.50 (0.01, 0.99)	0.04 (-0.62, 0.70)	0.9037
ROMs (mg H_2O_2/dL)	1.48 (0.25, 2.71)	2.26 (0.95, 3.57)	0.78 (-0.98, 2.54)	0.3831
8-isoprostane (pg/mL)	61.26 (43.99, 78.52)	44.82 (26.42, 63.22)	-16.44 (-41.14, 8.27)	0.1905

* Two-sided p-value <0.05 considered statistically significant. [a] Difference = supplementation – no supplementation; [b] Secondary outcomes shown are those with a significant change from Visit 1, calculated using LOCF method; [c] value based on n=64 observations. Calcidiol, 25-hydroxyvitamin D; DHA, docosahexaenoic acid; EPA, eicosapentaenoic acid; LSMEANS, least squares means; MMS, multiple micronutrient supplementation; RBC, red blood cells; ROMs, reactive oxygen metabolites; TFA, total fatty acids; wt, weight.

Table S6. Infant assessments (per protocol population; values expressed as mean ± standard deviation (range), unless otherwise indicated).

	No supplementation (N=76) [a]	MMS (N=65) [a]	Difference, LSMEANS (95% confidence interval) [b,c]	P-value
Gestational age (weeks)	39.9±1.20 (36.1-42.0)	39.9±1.12 (35.9-41.9)	-0.05 (-0.46, 0.35)	0.8019
Head circumference (cm)	34.4±1.08 (32.0-36.5)	34.4±1.14 (32.0-37.0)	-0.04 (-0.44, 0.35)	0.8250
Weight (kg)	3.4±0.43 (2.4-4.7)	3.4±0.41 (2.7-4.6)	-0.00 (-0.15, 0.14)	0.9542
Length (cm)	50.2±2.04 (45.0-56.0)	50.3±1.79 (45.7-54.3)	0.07 (-0.61, 0.76)	0.8346
Ponderal index (g/cm^3)	2.7±0.23 (2.1-3.5)	2.7±0.21 (2.0-3.1)	-0.03 (-0.11, 0.05)	0.4643
Skinfold thickness (mm)				
Triceps	4.2±0.80 (2.7-6.2)	4.4±0.86 (3.0-6.5)	0.19 (-0.13, 0.51)	0.2422
Biceps	3.5±0.66 (2.2-5.3)	3.54±0.70 (2.3-5.4)	0.03 (-0.23, 0.29)	0.8313
Suprailiac	4.4±0.94 (2.9-6.5)	4.6±0.92 (3.0-7.3)	0.18 (-0.17, 0.53)	0.3074
Subscapular	4.3±0.89 (2.6-6.7)	4.7±1.01 (3.0-8.4)	0.40 (0.04, 0.76)	0.0292 *
Bone density				
m^2	2990.9±161.62 (2688.0-3839.0)	2929.5±168.88 (2325.0-3215.0)	—	0.0486 *
%	24.8±25.03 (0.0-92.0)	20.3±21.22 (0.0-86.0)	—	0.3518
z-score	-0.9±0.99 (-3.7-1.4)	-1.2±1.41 (-6.4-2.4)	—	0.2936

* Two-sided p-value <0.05 regarded as statistically significant. [a] Number of subjects who performed the assessment varied for each parameter; [b] ANCOVA model adjusting for center; [c] difference = supplementation – no supplementation. LSMEANS, least squares means; MMS, multiple micronutrient supplementation.

Table S7. Daily macronutrient intakes during the study (per protocol population) compared with recommended allowances for pregnant women.

Macronutrients (units)	Current study, mean ± standard deviation (range)						IOM RDA [1]
	No supplementation (N=76)			MMS (N=65)			
	Visit 2	Visit 3	Visit 4	Visit 2	Visit 3 [a]	Visit 4	
SFA (g)	20.5 ± 6.8 (7-40)	20.2 ± 8.7 (7-52)	19.2 ± 7.3 (7-37)	21.4 ± 8.6 (9-67)	20.3 ± 8.1 (9-44)	19.6 ± 11.6 (7-85)	ALAP
DHA (g)	0.5 ± 0.4 (0-2)	0.5 ± 0.3 (0-2)	0.5 ± 0.4 (0-3)	0.4 ± 0.2 (0-2)	0.3 ± 0.2 (0-1)	0.4 ± 0.2 (0-1)	0.1 [3] or 0.2 [4] (not RDA)
EPA (g)	0.3 ± 0.2 (0-2)	0.3 ± 0.1 (0-1)	0.3 ± 0.2 (0-2)	0.2 ± 0.1 (0-1)	0.2 ± 0.1 (0-1)	0.2 ± 0.2 (0-1)	0.1 [3] or 0.2 [4] (not RDA)
Total proteins (g)	83.3 ± 33.5 (27-234)	78.1 ± 29.9 (25-201)	80.0 ± 31.9 (32-197)	76.2 ± 29.4 (31-203)	72.3 ± 25.4 (31-170)	74.1 ± 35.7 (30-213)	71
Carbohydrates (g)	287.7 ± 117.8 (150-675)	271.2 ± 125.8 (90-699)	273.4 ± 111.8 (106-689)	284.7 ± 123.6 (124-697)	265.8 ± 110.7 (73-628)	261.2 ± 122.6 (59-780)	175
Dietary fiber (g)	25.6 ± 13.5 (9-97)	23.0 ± 10.0 (8-64)	23.3 ± 10.8 (8-64)	23.1 ± 10.2 (7-53)	21.9 ± 9.2 (8-46)	21.3 ± 13.3 (3-79)	28 [b]

[a] N=64; [b] adequate intake (used when there isn't enough data to calculate an average requirement; the average nutrient level, based on observations or experiments, that is assumed to be adequate for the population's needs). Visit 2, baseline (GA week 13/15); Visit 3 (GA week 24/26); Visit 4 (GA week 34/36). ALAP, as low as possible; DHA, docosahexaenoic acid; EPA, eicosapentaenoic acid; IOM, Institute of Medicine; MMS, multiple micronutrient supplementation; RDA, recommended dietary allowance; SFA, saturated fatty acids.

References

1. Food Nutrition Board of the Institute of Medicine. Nutrient Recommendations: Dietary Reference Intakes (DRI). National Institutes of Health, Office of Dietary Supplements **2020**, https://ods.od.nih.gov/Health_Information/Dietary_Reference_Intakes.aspx. Date accessed: 1 Apr 2020.
2. Flock, M.R.; Harris, W.S.; Kris-Etherton, P.M. Long-chain omega-3 fatty acids: time to establish a dietary reference intake. *Nutr Rev* **2013**, *71*, 692-707.
3. Kris-Etherton, P.M.; Grieger, J.A.; Etherton, T.D. Dietary reference intakes for DHA and EPA. *Prostaglandins Leukot Essent Fatty Acids* **2009**, *81*, 99-104.
4. Koletzko, B.; Cetin, I.; Brenna, J.T. Dietary fat intakes for pregnant and lactating women. *Br J Nutr* **2007**, *98*, 873-877.
